# Posttraumatic osteoarthritis after athletic knee injury: A narrative review of diagnostic imaging strategies

**DOI:** 10.1002/pmrj.13217

**Published:** 2024-07-31

**Authors:** Alexandra E. Fogarty, Michael C. Chiang, Stephanie Douglas, Lauren H. Yaeger, Fabrisia Ambrosio, Christian Lattermann, Cale Jacobs, Joanne Borg‐Stein, Adam S. Tenforde

**Affiliations:** ^1^ Department of Anesthesia, Critical Care and Pain Medicine, Massachusetts General Hospital Harvard Medical School Boston Massachusetts USA; ^2^ Department of Physical Medicine and Rehabilitation, Spaulding Rehabilitation Hospital Harvard Medical School Charlestown Massachusetts USA; ^3^ Department of Orthopedic Surgery, Division of Physical Medicine and Rehabilitation Washington University School of Medicine St. Louis Missouri USA; ^4^ Becker Medical Library Washington University School of Medicine St. Louis Missouri USA; ^5^ Discovery Center for Musculoskeletal Recovery Schoen Adams Research Institute at Spaulding Boston Massachusetts USA; ^6^ Department of Orthopaedics, Brigham and Women's Hospital Harvard Medical School Boston Massachusetts USA; ^7^ Massachusetts General Brigham Sports Medicine, Brigham and Women's Hospital Harvard Medical School Boston Massachusetts USA

## Abstract

Intraarticular knee injuries and subsequent posttraumatic arthritis (PTOA) are common in athletes. Unfortunately, PTOA may significantly affect performance and overall function, but this condition remains difficult to characterize. In this review, we provide an overview of imaging modalities used to evaluate PTOA among athletes and physically active individuals following knee injury, with the goal to discuss the strengths and limitations of their application in this population. A literature search was performed to identify clinical studies focusing of knee injuries in athletes and athletic persons, specifically using imaging for diagnosis or monitoring disease progression. A total of 81 articles were identified, and 23 were included for review. Studies on plain radiographs (*n* = 8) and magnetic resonance imaging (MRI) assessed arthritic burden (*n* = 13), with MRI able to depict the earliest cartilage changes. Few studies (*n* = 2) leveraged ultrasound. Challenges persist, particularly regarding standardization and reliability across different radiographic grading systems. Additionally, further research is needed to establish the clinical significance of techniques to assess cartilage composition on MRI, including ultrashort echo‐time enhanced T2*, T1rho and T2 imaging. Addressing these challenges through standardized protocols and intensified research efforts will enhance the diagnostic utility of imaging modalities in musculoskeletal medicine and enable high‐quality prospective studies.

## INTRODUCTION

Posttraumatic osteoarthritis (PTOA) is a form of osteoarthritis that develops after joint injury such as anterior cruciate ligament (ACL) ruptures and meniscus tears, among others.^1^ It is characterized by cartilage degeneration, synovial inflammation, and bone changes. These injuries are common in athletes and can lead to early functional decline and pain, requiring earlier and more frequent interventions.^2^ ACL reconstruction is a common surgical procedure to improve function and knee stability, but ACL injury may lead to a 3‐fold increase in osteoarthritis compared to the contralateral knee, within 12–14 years after surgery.^3^ Meniscus tears are also a major independent risk factor for PTOA, with a prevalence of 21%–100% for patients with combined ACL and meniscal injuries.^4^ As a result, the burden of PTOA after ACL injury is substantial and suggests value of imaging criteria to detect disease burden that are both sensitive and specific.

The complex mechanisms that contribute to PTOA are not completely understood and may be distinct from primary arthritis. In primary OA, an abnormal cartilage matrix is affected by normal pressure forces. In contrast, PTOA may result from normal cartilage matrix subjected to an abnormal concentration of force across the joint.^5^ Joint trauma can directly damage cartilage and subchondral bone^6^; damage to either represents an independent risk factor for developing PTOA of the knee.^7^, ^8^ Joint trauma has been associated with the release of proinflammatory mediators, which peak 24 hours after ACL injury.^9^ These mediators activate biochemical pathways that culminate in chondrocyte apoptosis,^9^ alter gene expression in chondrocytes, and activate matrix metalloproteases.^10^ However, PTOA is increasingly recognized as a multifactorial process, involving a complex interplay between genetics, biological soundness, biomechanical health,^11^ body‐mass index,^12^ and psychological health.^13^

Athletes are more vulnerable compared to the general population to sustaining substantial intraarticular knee injuries, magnifying their predisposition to subsequent PTOA and its complexities.^14^ The importance of preventing sports‐related injury complications such as PTOA resonates across the entire athletic continuum, encompassing juvenile contenders, adult enthusiasts, and professionals and is similarly important within the sphere of military personnel. Professional athletes inhabit a unique landscape, amplifying their deviation from conventional scenarios.^15^ Given their reliance on sustained sports engagement for livelihood, the drive to hasten their reentry into competition remains conspicuous, possibly overshadowing considerations for long‐term joint health. The overarching objective revolves around the proficient diagnosis and management of this demographic, thereby optimizing both near‐term and enduring functional outcomes for all active persons.

The purpose of this narrative review is to describe the strength and limitations of imaging modalities used to describe PTOA in athletic populations. Use of valid, reliable, and disease‐specific objective clinical outcome measures may guide the diagnosis and treatment of PTOA in athletes and physically active individuals to ensure accurate diagnosis and management and assist with prognostication after injury. As the goal is to advance strategies to optimize nonsurgical care in younger populations, identifying reliable markers of disease represents an important first step.

## METHODS

### Eligibility criteria

Studies were included that described populations of athletes or athletic persons (defined as Tegner Activity Scale range 4–9) <50 years of age with cartilage loss and/or PTOA of the knee. Studies of patients with premorbid joint surgeries and/or studies of patients with rheumatologic conditions were excluded. There was no restriction on the type of imaging, outcomes measure, or follow‐up interval. We included both retrospective and prospective studies, but we excluded abstracts, communications, and other nonpublished literature. No publication date or language restrictions were imposed.

### Information sources and search

A medical librarian completed the primary search in Embase.com for the concepts of “post traumatic osteoarthritis,” “diagnostic imaging,” and “athletes” (referred to as the “primary search”). This search was run March 8, 2023, for keywords “PTOA AND (Dx AND imaging) AND athletes,” with no added limits. Article bibliographies were examined for additional references of interest and screening criteria were applied (six articles). Please see Appendix A for details of the full search strategy.

### Study selection and data items

Two authors independently assessed each abstract using screening criteria. Discrepancies were resolved by discussion in order to reach a final decision regarding study inclusion. Studies were organized by image modality. The following information was extracted from each study: author, year of publication. Information regarding grading paradigms, performance of each test (validity compared to arthroscopic standard, rater reliability, etc.) was described, if available.

## RESULTS

**FIGURE 1:**
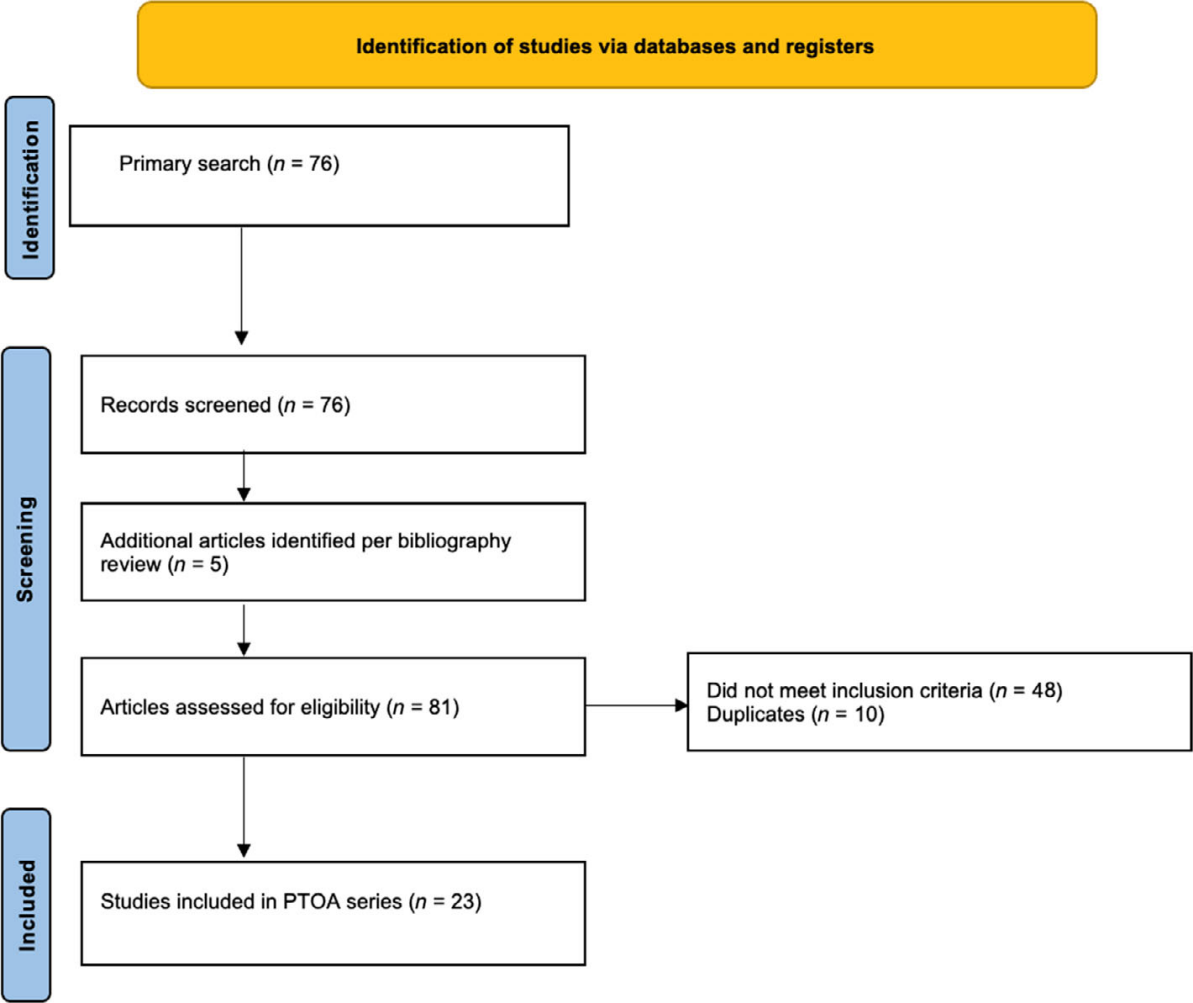
PRISMA diagram. PRISMA, Preferred Reporting Items for Systematic Reviews and Meta‐Analyses.

The search strategies revealed 76 results. Another five articles were considered for full‐text review after a bibliography screen identified relevant titles. After application of selection criteria, 23 articles were included for review (8 x‐ray [XR], 13 magnetic resonance imaging [MRI], 2 ultrasound [US]). Please see Figure 1 for Preferred Reporting Items for Systematic Reviews and Meta‐Analyses flow diagram for full list of included articles.

### Radiographs

We identified eight studies that used radiographs to characterize PTOA (Table 1). Participants most frequently had post‐ACL injury (seven studies). All studies used Rosenberg weight‐bearing anteroposterior views using either the Kellgren–Lawrence (KL) criteria (five studies),^4^, ^16^, ^17^, ^18^, ^19^ Osteoarthritis Research Society International (OARSI) criteria (three studies),^4^ International Knee Documentation Committee (IKDC) system (one study),^4^ or self‐described criteria (one study).^1^ Only one study directly investigated interrater reliability of radiographic criteria, with excellent agreement for the OARSI grading system (coefficient = 0.84), high agreement for IKDC system (0.71), and moderate for the KL system (0.48).^4^ When comparing radiographs to other imaging, the OARSI criteria had good correlation with magnetic resonance imaging (MRI) changes.^21^ On the other hand, KL graded radiographs were less sensitive compared to MRI (33%–39 used PTOA detection rate on MRI compared to 14%–21% on radiographs).^17^ Other studies in athletes have used radiographs with KL criteria to quantify posttraumatic arthritis severity but have not compared it to another grading system.^16^, ^19^

**TABLE 1:** Summary of imaging in knee PTOA using plain radiographs.

Author, year, PMID	Injury	Design	Modality	Reported results
Baumlein 2019, 30864087	Intraarticular tibial plateau fractures in skiers	Case series of athletes	XR (KL)	A longer period following surgery (*p* < .01) was linked with a more advanced stage of tricompartmental OA, as assessed at an average of 10.3 ± 1.9 years.
Fleming 2021,^20^ 32639610	ACL injury	RCT (ACLR with low graft tension versus ACLR with high graft tension)	XR (OARSI), MRI (WORMS)	The MRI WORMS indicated poorer results for the low‐tension group in comparison to the high‐tension group (*p* = .08).
Haberfield 2021, 33878493	ACL injury	Cohort study (return to sport *and* no return to sport)	MRI (MOAKS), XR (OARSI)	Reengaging in pivoting sports following ACL reconstruction is not linked to progression of OA on MRI (risk ratio range: 0.59–2.91).
Hoffelner 2012, 22265043	ACL injury	Cohort study (ACLR *and* contralateral knee)	MRI (ICRS), XR (KL)	There was no distinction in OA between the groups in either MRI or XR evaluations at an average follow‐up period of 10 years (MRI *p* = .64; XR *p* = .73).
MOON Group & Everhart 2021, 33793363	ACL injury	Case series	XR (OARSI, IKDC, KL)	The highest interrater reliability was observed for OARSI (Gwet's 0.84), with IKDC following closely (AC1 = 0.71), and KL exhibiting the lowest reliability (0.48). The 10‐year occurrence rate of clinical radiographic PTOA after ACL reconstruction is 37%.
Pedersen 2021, 34423060	ACL injury	Cohort study (isolated ACL *and* combined injury)	XR (KL)	There were no statistically significant variances in radiographic outcomes (*p* = .110–.919).
Smith 2017, 27940573	Intraarticular knee injuries in NFL football	Cohort study (history of injury and no history of injury)	XR, MRI: OA defined as (1) presence of joint space narrowing on XR or (2) evidence of moderate‐to‐severe nonfocal articular cartilage loss on MRI	OA was linked with surgery for a meniscal tear (*p* < .001), any past knee surgery (*p* < .001), or prior ACL reconstruction (*p* = .001).
Wellsandt 2018, 30505875	ACL injury	Cohort study (isolated ACL *and* combined injury)	XR (KL)	By 5 years 11.8% showed signs of OA in the medial compartment, whereas 88.2% did not. There was no significant differences (*p* < .05) between the operative and nonoperative groups.

Abbreviations: ACL, anterior cruciate ligament; ACLR, ACL repair; ICRS, International Cartilage Regeneration and Joint Preservation Society; IKDC, International Knee Documentation Committee; KL, Kellgren‐Lawrence; MOAKS, MRI Osteoarthritis Knee Score; MRI, magnetic resonance imaging; NFL, National Football League; OA, osteoarthritis; OARSI, Osteoarthritis Research Society International; PMID, PubMed identifier; PTOA, posttraumatic arthritis; RCT, randomized controlled trial; WORMS, Whole Organ Magnetic Resonance Imaging Score; XR, x‐ray.

### Magnetic resonance imaging (semiquantitative)

We identified seven studies that described using traditional MRI with semiquantitative grading (see Table 2). The most common injury was ACL injury, followed by meniscal lesions. When the performance of traditional MRI was compared to radiographs in athletes, traditional MRI identified osteoarthritis in 21% compared to 14% on radiographs using KL (*p* = .73).^17^ There is no clear consensus on which semiquantitative MRI grading method is superior in the athlete population with PTOA. All four of the semiquantitative grading systems have been used to study the incidence of PTOA in athletes in various contexts, including the MRI Osteoarthritis Knee Score (MOAKS; four studies), Whole Organ Magnetic Resonance Imaging Score (WORMS; one study),^22^ International Cartilage Regeneration and Joint Preservation Society (one study),^17^ and Modified Outerbridge Criteria (one study).^23^ No study directly compared the grading systems or against an arthroscopic standard.

**TABLE 2:** Summary of studies using MRI in knee PTOA.

Author, Year, PMID	Injury	Design	Modality	Reported results
Chu 2014, 24812196	ACL injury	Cohort study (ACLR *and* uninjured)	MRI UTE‐T2*	Preoperative increases in UTE‐T2 (*) values declined to levels comparable to those in uninjured controls (*p* = .02 and .005, respectively).
Haberfield 2021, 33878493	ACL injury	Cohort study (return to sport *and* no return to sport)	MRI (MOAKS), XR (OARSI)	Returning to pivoting sports post‐ACLR is not linked with a deterioration of osteoarthritis on MRI, with a risk ratio ranging from 0.59 to 2.91.
Hoffelner 2012, 22265043	ACL injury	Cohort study (ACLR *and* contralateral knee)	MRI (ICRS), XR (KL)	There was no variance in osteoarthritis between the groups on either MRI or XR at an average follow‐up duration of 10 years (MRI *p* = .64; XR *p* = .73).
Oak 2021, 33490295	ACL injury	Cohort study (ACLR *and* contralateral knee)	MRI (MOAKS)	There were no discernible variations in patellofemoral osteoarthritis between the cohorts after 2 years (*p* = .478).
Patterson 2020, 30762314	Intraarticular knee injuries	Cohort study (early ACLR *and* delayed ACLR and)	MRI (MOAKS)	MRI finding were not associated with patient outcomes. However, the presence of a patellofemoral cartilage defect at 1 year was significantly linked with a deterioration in KOOS‐Symptoms (*p* = .006).
Prien 2020 31209539	Intraarticular knee injuries in professional women's soccer	Cohort study (history of injury and no history of injury)	MRI (modified Outerbridge and Stoller classifications)	Partial meniscectomy for treating isolated meniscus injury was associated with osteoarthritis (OR = 5.4), but this association was not observed following isolated ACLR. Both meniscus and cartilage loss were predicted by injuries to the nonstriking leg (OR = 8.6, OR = 10.6), prior traumatic knee injury (OR = 4.6, OR = 2.6), and combined meniscus/ACL injuries (OR = 14.8, OR = 9.5).
Su 2013, 23707754	ACL injury	Cohort study (ACLR *and* uninjured)	MRI (T1 Rho, T2)	At baseline, T1Rho values were notably elevated in ACL‐injured knees, and these values exhibited a significant increase over the course of 2 years (*p* < .001).
Titchenal 2018, 29293364	ACL injury	Cohort study (ACLR *and* uninjured)	MRI UTE‐T2*	In ACLR, there was a positive correlation between UTE‐T2* values of the central medial femoral condyle cartilage and increasing varus alignment (*R* = 0.568).
Wang 2016, 26620091	ACL injury	Cohort study (ACL injury *and* contralateral knee)	MRI (T1 Rho, T2)	Knees with ACL injuries but no meniscal tears showed elevated T1Rho values compared to contralateral knee, (*p* = .002) and knees without injuries (*p* = .006). The average T2 values were greater in ACL‐injured knees without meniscal tears compared to knees without injuries (*p* = .001).
Welsh 2022, 35182170	Intraarticular knee injuries in professional men's soccer	Cohort study (history of injury and no history of injury)	MRI (WORMS)	The WORMS was associated with both the number of days missed due to injury (r: 0.489, *p* < .001) and previous knee injuries (r: 0.424, *p* < .001).
Whittaker 2016, 29018061	Intraarticular knee injury	Cohort study (knee injury *and* age‐matched controls)	MRI (MOAKS)	The knee injury group exhibited a 10‐time increase in the likelihood of developing osteoarthritis compared to uninjured participants at the 10‐year mark (*p* < .001).
Williams 2018, 29426012	Status post ACLR	Cohort study (ACLR *and* uninjured)	MRI UTE‐T2*	UTE‐T2* values displayed a moderate correlation with increased knee adduction moments (*r* = 0.41, *p* < .015).
Williams 2019, 30030866	Status post ACLR	Cohort study (ACLR *and* uninjured)	MRI UTE‐T2*	ACLR exhibited persistent elevations in T2* values in the deep cartilage of the medial tibiofemoral compartment compared to controls (*p* = .007).

Abbreviations: ACL, anterior cruciate ligament; ACLR, anterior cruciate ligament repair; ICRS, International Cartilage Regeneration and Joint Preservation Society; KL, Kellgren–Lawrence; KOOS, Knee Injury and Osteoarthritis Outcome Score; MOAKS, MRI Osteoarthritis Knee Score; MRI, magnetic resonance imaging; OARSI, Osteoarthritis Research Society International; OR, odds ratio; PMID, PubMed identifier; PTOA, post‐traumatic arthritis; UTE‐T2*, ultrashort echo‐time enhanced T2*; WORMS, Whole Organ Magnetic Resonance Imaging Score; XR, x‐ray.

### Magnetic resonance imaging (quantitative/compositional)

An additional two studies described using quantitative MRI with T1Rho and T2 (see Table 2). In a cohort study of young athletes with ACL injuries, participants underwent prerepair imaging, including XR with KL grading and quantitative MRI with T2 and T1Rho and imaging was repeated at 1 and 2 years after ACL repair.^24^ With regard to T1Rho, values were significantly higher in ACL‐injured knees at baseline and increased over the 2‐year study compared to that of the control knees. Similar findings were seen with T2. In the patients with the highest baseline KL scores, greater T1Rho signal was noted in all compartments at baseline, and a higher rate of change in T1Rho signal was observed from baseline to 2 years. This observation suggested that more baseline OA defined using KL classification combined with greater T1Rho signal may each correlate with higher rate of cartilage degradation after ACL injury.^24^

### Ultrasound

We identified two studies that describe using US to define and follow PTOA progression in athletes with knee injuries (See Table 3). In the first study,^25^ preseason ultrasonography of rugby players revealed greater lateral and medial femoral condylar thickness (*p* = .02 and *p* = .03) compared with postseason, regardless of injury history. Of note, those with prior injury were found to have greater lateral condylar thickness (*p* = .03), intercondylar thickness (*p* = .03), and partial area (*p* = .02) compared to control players at baseline. Similarly, in a cohort study of athletic persons with ACL repair, the index knee showed greater anterior femoral cartilage cross‐sectional area compared to both the uninjured (*p* = .04) contralateral (*p* = .001) limbs in athletic persons who were 37.0 ± 26.6 months from surgery.^26^ However, unlike the prior study, it is not specified if these persons were still participating in sports and only a single time point was described. Authors discuss that increased cartilage thickness in the initial phase post‐injury likely represents a pathologic change in cartilage morphology. Although the traditional paradigm imparts that joint space narrowing defines OA, early stages may be defined by cartilage thickening followed by thinning of the cartilage, which has been described in cohorts of primary knee OA on MRI.^27^

**TABLE 3:** Summary of ultrasound in knee PTOA.

Author, year, PMID	Injury	Study design	Modality	Reported results
Hori 2021, 34312456	Intraarticular knee injuries in rugby players	Cohort study (knee injury *and* age‐matched controls)	Ultrasound	Before the season, ultrasonography revealed that lateral condylar thickness (*p* = .02) and medial condylar thickness (*p* = .03) were greater than the measurements taken after the season, irrespective of injury history. Individuals with previous injuries showed increased lateral condylar thickness (*p* = .03), intercondylar thickness (*p* = .03), and partial area (*p* = .02) compared to control players.
Harkey 2018, 30615493	ACL injury	Cohort study (ACLR and age‐matched controls)	Ultrasound	In athletic individuals compared to controls, knees that underwent ACL reconstruction (ALCR) exhibited a larger anterior femoral cartilage cross‐sectional area compared to both the contralateral (*p* = .001) and uninjured (*p* = .04) limbs. This assessment was made at a time point approximately 37.0 ± 26.6 months after surgery.

Abbreviations: ACL, anterior cruciate ligament; ACLR, anterior cruciate ligament repair; PMID, PubMed identifier; PTOA, posttraumatic arthritis.

## DISCUSSION

**FIGURE 2:**
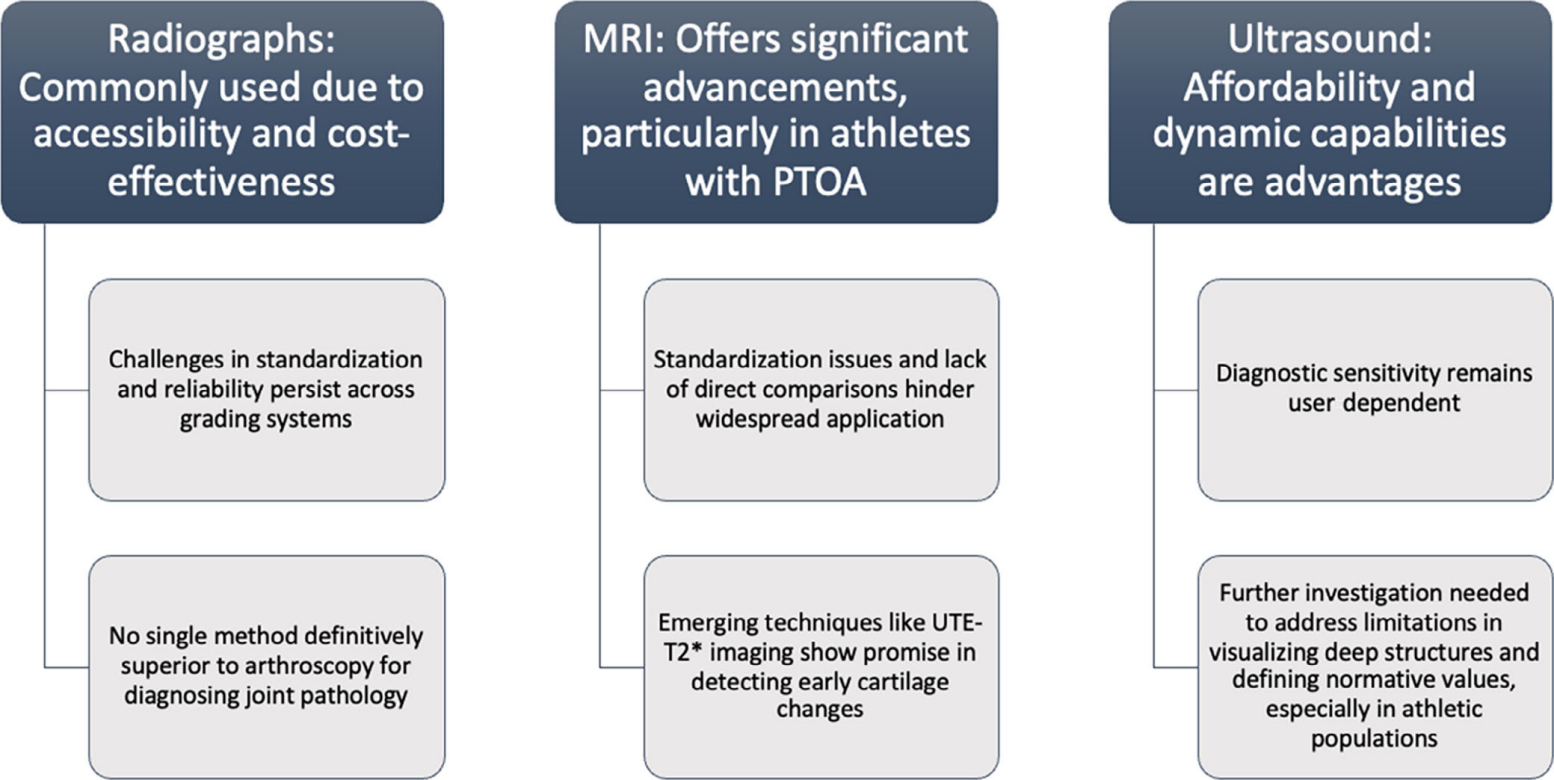
Summary of imaging modalities in osteoarthritis and post‐traumatic arthritis diagnosis. MRI, magnetic resonance imaging; PTOA, posttraumatic arthritis; UTE‐T2*, ultrashort echo‐time enhanced T2*.

In this review, we aim to discuss the advantages and limitations of various imaging modalities in the context of diagnosing PTOA in athletes and athletic persons. A total of 76 results and 23 articles were selected for review, encompassing 8 studies using XR, 13 employing MRI, and 2 using US as imaging modalities. This review aims to delve into the advantages and limitations of various imaging modalities in diagnosing PTOA among athletes and athletic individuals. Please refer to Figure 2 for overview of common themes.

### Radiographs

Radiographs remain the gold standard for the diagnosis of both OA and PTOA.^28^ Benefits of radiographs include low cost and accessibility, low radiation dose, and ability to perform weight‐bearing exams. However, the two‐dimensional nature, low sensitivity to early joint changes, lack of soft tissue visualization, and potential for projection errors are important limitations.

There are several classification systems that categorize the severity of osteoarthritis on plain radiographs. The most frequently cited is the 5‐point semiquantitative KL scale first described in 1957.^29^ Its use has been validated for multiple joints against direct cartilage visualization by arthroscopy and through cadaver dissection.^30^, ^31^ Newer grading systems have emerged, including the OARSI criteria IKDC criteria, among others.^31^, ^32^ Although none of these systems were designed exclusively for PTOA in athletes, several systems have been applied to this population and are discussed in depth in a subsequent section.

There is limited evidence to support the use of a single grading criteria. In athletes with PTOA, the single head‐to‐head study of radiographic methods favored the use of OARSI criteria but did not compare its utility against MRI or arthroscopy‐based diagnosis. When comparing each radiographic diagnosis against arthroscopy in knees, authors have shown that there is a low level of correlation between radiographic grading and cartilage degeneration, with no single grading system statistically outperforming another.^30^, ^31^ Such data have led to many experts concluding that current grading systems are largely equivocal for primary knee OA.^33^ In athletes with PTOA, the single head‐to‐head study of radiographic methods also favored the use of OARSI criteria but did not compare their utility against MRI or arthroscopy‐based diagnosis.

Intra‐ and interrater reliability has challenged the application of radiographic criteria. In a Multicenter ACL Revision Study (MARS) consortium study of primary knee OA in patients undergoing ACL revision, six radiographic classification systems were assessed for interrater reliability and compared with arthroscopy.^33^ The IKDC system performed the best, with interobserver reliability rated as “good” (coefficient of 0.59 and 0.66 on anteroposterior and Rosenberg view respectively), compared with “moderate” (0.38 and 0.54) using KL method. Out of the six classification systems, no method showed “very good” interrater reliability for classifying tibiofemoral osteoarthritis (defined as correlation coefficient of 0.8–1.0). Similarly, the OARSI‐OMERACT (Outcome Measures in Rheumatology) task force studied the reliability of three commonly used systems to grade knee OA. Authors concluded that no one method was highly superior to the other in terms of rater reliability. Joint space narrowing, particularly in knee flexed view, appears to be the most reliably identified feature (kappa, 0.86 vs. 0.56 and 0.48 for KL and OARSI, respectively).^34^

Poor standardization of x‐ray views is a contributor to poor rater reliability. Many authors have argued that optimization of images greatly improves the sensitivity of diagnostic criteria.^35^ For example, posterior–anterior radiographs with weight‐bearing and 45° of knee flexion, also known as the Rosenberg view, is now considered the gold standard view in knee arthritis assessment. Rosenberg radiograph had a better correlation in predicting total chondral disease on arthroscopy (Spearman rho = 0.36; 95% confidence interval [CI], 0.32–0.39) versus anteroposterior radiograph (Spearman rho = 0.29; 95% CI, 0.26–0.32).^33^ Rosenberg view may also depict severity of disease in the lateral compartment and joint space narrowing in patellofemoral OA.^36^, ^37^, ^38^ Imaging optimization tools such as rigid frames help improve the consistency of patient positioning in clinical trials.^39^ Although no study in our review featured the use of such a positioning device, some societies now recommend their use to improve standardization.^40^ Limitations to image quality and rater training contribute to the wide range of prevalence estimates for PTOA.^41^ Nonetheless, standardization, use of posterior–anterior radiographs, and tutorials for raters are likely important when designing future studies of athletes with PTOA.

### Magnetic resonance imaging

MRI has emerged as the diagnostic modality of choice to diagnose early knee arthritis changes. MRI stimulates protons by magnetic fields to deliver high‐quality, two‐ or three‐ dimensional images^42^ and allows for visualization of intraarticular structures such as cartilage.^43^ Despite these advantages, MRI is expensive, time consuming, requires specific protocolization, and its use is limited by metal implants.^44^ Arthritic changes on MRI can be assessed using semiquantitative grading methods, which have been validated in large studies.^43^, ^45^ Quantitative MRI, also known as compositional MRI, enables the earliest detection of prearthritic changes.^43^ Similarly, MRI‐based 3D bone shape is emerging as a means of predicting the onset of radiographic OA, but its use remains experimental.^46^

Traditional MRI relies on semiquantitative scoring methods to grade the severity of imaging findings.^47^ WORMS is the first grading tool that was developed.^47^ It has been applied in a multitude of large OA studies and has shown high interrater reliability (correlations >0.8).^48^, ^49^ Other important scoring systems emerged thereafter, notably the Boston Leeds Osteoarthritis Knee Score (BLOKS) and the MOAKS, which merged strategies from WORMS and BLOKS.^50^ Notably, in studies comparing 115 knees with radiographic OA per WORMS and BLOKS, the two methods had good interreader agreement for all features.^51^ However, WORMS performed better for predicting later cartilage loss compared to BLOKS. On the other hand, BLOKS included a more detailed analysis of meniscal lesions, including signal abnormality and uncommon types of tears.^51^, ^52^ MOAKS combines the strengths of the two systems, by refining the scoring of bone marrow lesions and enhancing the elements of meniscal morphology and is the accepted standard. MOAKS has not been directly compared to WORMS or BLOKS. All measures of rater reliability using kappa statistics were strong (0.61–1.0).^53^

All of the semiquantitative grading systems have been used to study the incidence of PTOA in athletes in various contexts. MOAKS was used to evaluate PTOA incidence in athletes returning to pivoting sport after ACL injury, showing that return to play was not associated with worsening of MRI features.^21^ Similarly, WORMS has also been used in a study of young athlete knee injuries, where a higher score was significantly correlated with a prior knee injury of any kind (r: 0.424, *p* < .001).^22^ Prien et al. employed the modified Outerbridge and Stoller classifications to look at chondral lesions longitudinally in professional soccer athletes and found an association with higher grades and those with prior partial meniscectomy for isolated meniscus injury (odds ratio [OR] = 5.4), but not following isolated ACL injury with reconstruction.^23^ The combination of meniscus with ACL injuries was associated with highest risk for meniscus and chondral loss (OR = 14.8, OR = 9.5, respectively). There is no clear consensus on which semiquantitative MRI grading method is superior in the athletic population with PTOA. In addition, the Anterior Cruciate Ligament OsteoArthritis Score (ACLOAS) is a novel MRI‐based scoring system to assess acute ACL injury and longitudinal osteoarthritis changes, whose use we anticipate increasing in future research. Its validation in athletic cohorts is still needed.^54^

A whole‐joint semiquantitative grading system was specifically developed to assess longitudinal changes after ACL injury.^55^ The grading system involves assessing the following joint features: condition of the cruciate and collateral ligaments, condition of the ACL graft postoperatively, meniscus morphology/extrusion, osteophytes, traumatic and degenerative bone marrow lesions, osteochondral injury, Hoffa synovitis, and effusion synovitis. Of the 142 parameters assessed, 73% showed w‐kappa values between 0.80 and 1.00 and 92% showed agreement above 80%. Intraobserver reliability ranged between 0.52 and 1.00 and interobserver reliability ranged between 0.00 and 1.00. The highly variable interobserver reliability was suggested to be related to the low frequency of some of the features. This system has been used to assess the role of persistent synovitis on OA progression,^56^ ACL healing after nonoperative treatment,^57^ and whether early surgical treatment was protective against secondary meniscus injury compared to conservative treatment.^58^

Not only are changes in the articular surface noted after ACL injury, early changes in bone shape have also been reported, including alterations in femoral condyle morphology, tibial plateau area, and tibial slope, which can be detected on MRI within 1–3 years following ACL injury or reconstruction.^59^, ^60^ Medial femoral condyle bone area increases have been observed the first few months following ACL reconstruction.^60^, ^61^ Notably patient‐reported outcomes and features of cartilage quality using MRI at 3 years correlated with changes of bone shape observed within 6 months of ACL reconstruction.^61^ Interestingly, the magnitude of bone surface increase was greater for those with concomitant meniscal injury.^58^ These results are clinically meaningful as the changes in bone shape precede articular cartilage changes.

Ultrashort echo‐time enhanced T2* (UTE‐T2*) has been proposed as a clinically relevant MRI tool for detecting early changes in cartilage following athletic injury. Among the 13 MRI studies in athletes with PTOA, 4 describe using MRI UTE‐T2* (see Table 2). In a study of 38 ACL reconstructed knees, patients underwent MRI UTE‐T2* imaging of the medial tibial plateau and femoral cartilage. Approximately half of the patients demonstrated increased T2* in the medial tibiofemoral deep cartilage, with values more than 2 SDs higher than uninjured controls.^62^ Another study of ACL injured patients showed that T2* mapping is sensitive to deep cartilage changes reflective of acute injury as well as signs of early degeneration. Interestingly, the return of elevated T2* values in post‐ACLR patients to similar measures in healthy controls might indicate potential for healing.^62^ Recently, the clinical relevance of T2* changes have also been investigated. T2* change correlated with knee adduction moment following ACLR.^63^, ^64^

### Ultrasound

US uses high‐frequency sound waves to image tissues with broad application in musculoskeletal medicine.^65^ It is estimated to be less expensive, better tolerated by patients, available for point of care applications, and able to function as a dynamic tool that can be tailored to the specific clinical situation. However, the diagnostic sensitivity in musculoskeletal medicine is highly user dependent. It is also unable to visualize deep structures obscured by bone or fluid layers.^66^

Few studies have evaluated diagnosis of osteoarthritis when comparing US to conventional radiography. In a study of patients with primary knee osteoarthritis, the diagnostic properties of US were evaluated using x‐ray as a reference method. Osteophyte presence or femoral cartilage thickness loss had the best sensitivity of 95%, and the combined finding of osteophyte and femoral cartilage thickness loss had the best specificity at 94%.^67^ In a study looking at the relationship between radiographs with KL grading and sonographic knee arthritis, there was a good association between number of sonographic findings and radiographic KL grade.^68^ Another study looked at the association between articular cartilage surface integrity assessed by US and reported association between sonographic and histopathological features.^69^ Although this literature appears promising, studies are small.

When compared with MRI as the reference standard, US appears to have limitations. US has been estimated to image only approximately 66% of the medial femoral condyle (two thirds) and 37% of the lateral femoral condyle cartilage (one third), compared with MRI, which has the ability to visualize nearly 100% of the articular cartilage.^70^ In one study comparing US to MRI visualization of femoral cartilage pathology, US resulted in underestimation of the changes in thickness by 21%–25% compared to MRI.^70^ Another possible limitation of the use of US for diagnosis is the user variability, which has been described in other US applications. However, studies of primary knee arthritis have found good intra‐ and interrater reliability for arthritic changes, particularly for joint effusions, cartilage loss, medial meniscal damage, Baker's cysts, and presence of osteophytes.^71^, ^72^ Finally, another limitation stems from poorly defined normative values.^70^ These values are likely different in healthy athletes, who have been shown to have thicker femoral cartilage than sedentary individuals (*p* < .001).^73^

## CONCLUSION

In conclusion, studies of athletic individuals leverage imaging for the diagnosis of PTOA differently. Although radiographs remain a common diagnostic tool due to their accessibility and cost‐effectiveness, challenges persist in standardization, reliability across different grading systems, and the ability to detect early PTOA changes. Despite efforts to optimize views, no single radiographic method has been definitively proven superior for diagnosing joint pathology compared to arthroscopy. Meanwhile, MRI offers significant advancements in diagnosing knee arthritis, particularly in athletes with PTOA, yet standardization issues and the lack of direct comparisons between grading systems hinder its widespread application. Emerging compositional MRI techniques like UTE‐T2*, T1rho, and T2 imaging show promise in detecting early cartilage changes, but further research is needed to establish their clinical significance. Similarly, although US presents advantages such as affordability and dynamic capabilities, its diagnostic sensitivity remains user dependent, and limitations in visualizing deep structures and defining normative values warrant further investigation, especially in athletic populations. Overall, addressing these challenges through standardized protocols and increased research efforts will enhance the diagnostic utility of these imaging modalities in musculoskeletal medicine.

## DISCLOSURES

Adam Tenforde serves as senior editor for *PM&R Journal*. He gives professional talks such as grand rounds and medical conference plenary lectures and receives honoraria from conference organizers. He has participated in research funded by Arnold P. Gold Foundation (physician and patient care disparities), Football Player Health Study at Harvard (health in American‐Style Football players), American Medical Society for Sports Medicine (bone density research), Uniform Health Service and Enovis (Achilles tendinopathy), and MTEC/Department of Defense (bone stress injuries with shockwave). He is a paid consultant for State Farm Insurance and Strava.

## Supporting information


**Data S1.** Supporting Information.
