# Supplementary material for: Posttraumatic osteoarthritis after athletic knee injury: A narrative review of diagnostic imaging strategies
Source: PM R. 2024 Jul 31;17(1):96–106. doi: 10.1002/pmrj.13217 (PMC11733860; doi:10.1002/pmrj.13217)
Supplement: Supplementary file 1 — Data S1. Supporting Information. [file PMRJ-17-96-s001.docx]

**APPENDIX: SEARCH STRATEGY**

**Primary search**

Embase

PTOA AND (Dx AND imaging) AND athletes

76 results 3/8/23

('post traumatic osteoarthritis'/exp OR ((('post-trauma*' OR 'posttrauma*' OR postramua*) NEAR/3 (osteoarthritis OR oa)):ti,ab,kw) OR (((trauma- OR trauma*) NEAR/3 'joint degeneration'):ti,ab,kw)) AND (('diagnostic procedure'/de OR 'diagnosis'/de) AND ('computer assisted tomography'/de OR 'positron emission tomography'/de OR 'nuclear magnetic resonance imaging'/de OR 'imaging'/de OR 'optical coherence tomography'/de OR 'radiodiagnosis'/de OR 'echography'/de OR 'ultrasound'/de) OR (diagnos*:ti,ab,kw AND (grading:ti,ab,kw OR imaging:ti,ab,kw OR 'bone scan bone scintigraphy':ti,ab,kw OR cat:ti,ab,kw OR pet:ti,ab,kw OR eos:ti,ab,kw OR 'il-6':ti,ab,kw OR mri:ti,ab,kw OR mei:ti,ab,kw OR 'optical coherence tomography':ti,ab,kw OR oct:ti,ab,kw OR 'x ray':ti,ab,kw OR 'rosenberg radiograph':ti,ab,kw OR doptone:ti,ab,kw OR sonographic:ti,ab,kw OR sonogram:ti,ab,kw OR ultrasonic*:ti,ab,kw OR echography:ti,ab,kw OR echogram:ti,ab,kw OR echographic:ti,ab,kw OR echoscopy:ti,ab,kw OR echosound:ti,ab,kw OR ultrasono*:ti,ab,kw OR 'tnf-alpha':ti,ab,kw OR sonication:ti,ab,kw OR sonification:ti,ab,kw OR 'ultra sound':ti,ab,kw OR ultrashell:ti,ab,kw OR xr:ti,ab,kw OR ((compute* NEAR/3 tomography):ti,ab,kw) OR (((positron OR magnetic) NEAR/3 tomograph*):ti,ab,kw) OR (((radio*hic OR radiology OR radio OR roentgen OR roentgenologic OR roentgenologic) NEAR/3 (diagnos* OR examination OR screening)):ti,ab,kw))) OR 'kellgren-lawrence grade'/exp OR 'international knee documentation committee'/exp OR 'international knee documentation committee scale'/exp OR 'boston leeds osteoarthritis knee score'/exp OR 'hip disability and osteoarthritis outcome score'/exp OR 'knee injury and osteoarthritis outcome score'/exp OR 'patient reported outcomes measurement information system'/exp OR ((kellgren NEAR/2 lawrence):ti,ab,kw) OR ((('hip disability' OR 'hip dysfunction') NEAR/2 'osteoarthritis outcome score'):ti,ab,kw) OR ((knee NEAR/2 ('osteoarthritis outcome score' OR 'osteoarthritis outcome scale')):ti,ab,kw) OR 'kellgren-lawrence':ti,ab,kw OR 'kl classification':ti,ab,kw OR 'kl scoring':ti,ab,kw OR orasi:ti,ab,kw OR 'oarsi-omerac':ti,ab,kw OR 'international knee documentation committee':ti,ab,kw OR ikdc:ti,ab,kw OR 'tonnis criteria':ti,ab,kw OR 'ahlback scale':ti,ab,kw OR 'brandt scale':ti,ab,kw OR 'cal scale':ti,ab,kw OR 'osteoarthritis computed tomography':ti,ab,kw OR 'ultrasonographic grading scale':ti,ab,kw OR 'knee osteoarthritis scoring system':ti,ab,kw OR koss:ti,ab,kw OR 'boston leeds osteoarthritis knee score':ti,ab,kw OR bloks:ti,ab,kw OR hoos:ti,ab,kw OR 'hip osteoarthritis outcome score':ti,ab,kw OR koos:ti,ab,kw OR 'patient-reported outcomes measurement information system':ti,ab,kw OR promis:ti,ab,kw) AND ('athlete'/exp OR 'return to sport'/exp OR 'sport'/de OR 'sport injury'/exp OR 'athletic performance'/exp OR 'baseball'/exp OR 'baseball player'/exp OR athlet*:ti,ab,kw OR sportman:ti,ab,kw OR sportsmen:ti,ab,kw OR sportspeople:ti,ab,kw OR sportsperson*:ti,ab,kw OR sportsplayer*:ti,ab,kw OR sportswoman:ti,ab,kw OR sportwomen:ti,ab,kw OR 'return to sport*':ti,ab,kw OR 'resumption of sport*':ti,ab,kw OR 'return to play*':ti,ab,kw OR baseball:ti,ab,kw OR (((athletic OR athlete OR sport*) NEAR/3 (injur* OR trauma OR accident* OR performance)):ti,ab,kw))
